# Evaluation of ABT-751, a novel anti-mitotic agent able to overcome multi-drug resistance, in melanoma cells

**DOI:** 10.1007/s00280-023-04624-6

**Published:** 2024-01-16

**Authors:** Thamir M. Mahgoub, Emmet J. Jordan, Amira F. Mahdi, Veronika Oettl, Stefanie Huefner, Norma O’Donovan, John Crown, Denis M. Collins

**Affiliations:** 1 Cancer Biotherapeutics Research Group, School of Biotechnology, Dublin City University, Glasnevin, Dublin 9, Ireland; 2 School of Medicine, University of Limerick, Limerick, Ireland; 3 Department of Medical Oncology, St Vincent’s University Hospital, Elm Park, Dublin 4, Ireland

**Keywords:** Melanoma, Multi-drug resistance, P-gp, ABT-751, Taxanes, BCRP, MDR3

## Abstract

**Purpose:**

Drug efflux transporter associated multi-drug resistance (MDR) is a potential limitation in the use of taxane chemotherapies for the treatment of metastatic melanoma. ABT-751 is an orally bioavailable microtubule-binding agent capable of overcoming MDR and proposed as an alternative to taxane-based therapies.

**Methods:**

This study compares ABT-751 to taxanes in vitro, utilizing seven melanoma cell line models, publicly available gene expression and drug sensitivity databases, a lung cancer cell line model of MDR drug efflux transporter overexpression (DLKP-A), and drug efflux transporter ATPase assays.

**Results:**

Melanoma cell lines exhibit a low but variable protein and RNA expression of drug efflux transporters P-gp, BCRP, and MDR3. Expression of P-gp and MDR3 correlates with sensitivity to taxanes, but not to ABT-751. The anti-proliferative IC_50_ profile of ABT-751 was higher than the taxanes docetaxel and paclitaxel in the melanoma cell line panel, but fell within clinically achievable parameters. ABT-751 IC_50_ was not impacted by P-gp-overexpression in DKLP-A cells, which display strong resistance to the P-gp substrate taxanes compared to DLKP parental controls. The addition of ABT-751 to paclitaxel treatment significantly decreased cell proliferation, suggesting some reversal of MDR. ATPase activity assays suggest that ABT-751 is a potential BCRP substrate, with the ability to inhibit P-gp ATPase activity.

**Conclusion:**

Our study confirms that ABT-751 is active against melanoma cell lines and models of MDR at physiologically relevant concentrations, it inhibits P-gp ATPase activity, and it may be a BCRP and/or MDR3 substrate. ABT-751 warrants further investigation alone or in tandem with other drug efflux transporter inhibitors for hard-to-treat MDR melanoma.

## Introduction

Metastatic melanoma carries a poor prognosis with a 5-year survival rate of 31.5% and an age-adjusted mortality rate of 2 in 100,000 people [1]. Furthermore, incidence of melanoma is set to increase, with GLOBOCAN predicting a 57% increase in melanoma diagnoses worldwide by 2040 [2]. The standard of care regime for metastatic melanoma without BRAF mutation is immunotherapy with the immune checkpoint inhibitors pembrolizumab, ipilimumab and nivolumab or a combination thereof [3]. However, for patients who are deemed ineligible for immunotherapy, or who do not harbour a targetable mutation such as BRAF, chemotherapy is still the main option. Response rates are approximately 20% for single agent chemotherapy [4], with combination regimens offering little improvement in response alongside the additional toxicity [5]. The intrinsically refractory nature of melanoma means more chemotherapeutic options are always needed to prolong the survival of patients with hard-to-treat tumours.

The taxanes, such as docetaxel and paclitaxel, are a class of agents that have shown efficacy in multiple cancer types alone and in combination with other chemotherapeutics. Their principal mechanism of action is the disruption of the microtubule function and inhibiting mitotic spindle dynamics, leading to mitotic arrest and cell death [6]. While taxanes inhibit melanoma cell growth at nanomolar concentrations in vitro, this effect does not translate to the clinical setting where single agent taxane efficacy has been limited and combination therapy results variable in numerous clinical trials, as reviewed in [7].

A postulated mechanism of in vivo resistance to taxanes in melanoma is the action of drug efflux transporter proteins, chiefly P-glycoprotein (P-gp/multi-drug resistance protein 1/MDR1/ABCB1). P-gp is a human ABC-transporter of the MDR/TAP subfamily that transports various substrates out of cells including taxanes, BRAF inhibitors (vemurafenib, dabrafenib, encorafenib) and MEK inhibitors (trametinib, cobimetinib, binimetinib) and numerous other cancer therapies [8–11]. P-gp was reported to be expressed in 33% (11/33) of a melanoma cell line panel derived from primary and metastatic malignant melanoma lesions [12]. Previous studies in our laboratory have shown that P-gp is detected in approximately 83% (158/191) of melanoma tissue samples [13]. Multi-drug resistance protein 3 (MDR3/ABCB4) is a sister protein of P-gp, with high sequence homology between the two genes encoding the proteins [14]. Increased MDR3 expression is seen in models of acquired taxane resistance across cancer types and MDR3 has been reported to play an active, complementary role to P-gp in mediating this resistance [15, 16]. Breast cancer resistance protein 1 (BCRP/ABCG2) is a drug efflux ABC-transporter with complementary action to P-gp, which is associated with resistance to many cancer therapies [17] and also reportedly expressed in melanoma tissue and cell lines [18, 19].

ABT-751 is an orally bioavailable anti-mitotic sulphonamide, considered a second generation microtubule-binding agent. ABT-751 binds to the colchicine-binding site on β-tubulin and inhibits the polymerization of microtubules, thereby preventing tumour cell replication [20]. ABT-751 has been investigated in a number of malignancies including liver (NCT00073112), non-small cell lung (NCT00073151), breast (NCT00073138) and colorectal cancers (NCT00073138). Studies in the lung cancer setting have reported acceptable toxicity profiles and modest anti-tumour activity for ABT-751, both alone [21] and in combination with platinum-based chemotherapy [22], with further investigation on biomarkers and potential therapeutic combinations recommended. It has also undergone phase I and II trials for paediatric neuroblastoma (NCT00436852), which found ABT-751 to be well tolerated but not associated with increased objective response rate or time to progression [23]. Recent pre-clinical studies have examined ABT-751 as a potential treatment for urinary bladder urothelial carcinoma and p53-deficient hepatocellular carcinoma [24, 25]. ABT-751 has a similar mechanism of action to the taxanes, but while the taxanes are substrates for P-gp, a study using an in vivo xenograft model suggests that ABT-751 is not [26] and can overcome P-gp-mediated resistance. This would suggest higher concentrations of ABT-751 may be achieved within cells that express P-gp, and that this compound may be beneficial in multi-drug resistant taxane refractory melanoma [27].

We examined the sensitivity of seven melanoma cell lines to ABT-751 and the taxanes, paclitaxel and docetaxel, and investigated the relationship between drug efflux transporter expression and drug sensitivity. We also examined the effects of the P-gp/BCRP inhibitor elacridar on response to ABT-751 in a P-gp over-expressing cell line model, DLKP-A, as well as melanoma models of varying drug efflux transporter expression. Lastly, we profiled the direct interaction between ABT-751 and P-gp and BCRP using cell-free ATPase assays. The purpose of this study was to explore the interaction between ABT-751 and drug efflux transporter proteins and assess the potential suitability of ABT-751 for the treatment of multi-drug resistant (MDR) melanoma.

## Methods

### Cells and reagents

Lox-IMVI, Malme-3M, Sk-Mel-5, and Sk-Mel-28 were obtained from the Department of Developmental Therapeutics, National Cancer Institute (NCI). WM-115, WM-266-4 were obtained from the European Collection of Authenticated Cell Cultures (ECACC) and the SK-Mel-2 cell line was obtained from the American Type Culture Collection (ATCC). Cell lines were maintained at 37 °C with 5% CO2 in RPMI-1640 medium (Sigma) with 10% FCS (BioWhittaker). DLKP-A is an adriamycin-resistant, P-gp over-expressing variant of DLKP, established in the National Institute for Cellular Biotechnology from a squamous cell lung carcinoma sample [28]. DLKP-Mitox is a mitoxantrone-resistant sub-variant of DLKP that over-expresses BCRP that was used as a positive control for BCRP expression also established in the National Institute for Cellular Biotechnology [29]. DLKP-A, DLKP-Mitox and DLKP cells were maintained in DMEM/Ham’s F12 1:1 medium (Sigma) with 5% FCS (BioWhittaker). Stock solutions of ABT-751 (10 mM) (Abbott), and elacridar (3.56 mM) (Sigma-Aldrich) were prepared in dimethyl sulfoxide (Sigma-Aldrich). Clinical formulations of docetaxel (11.6 mM) and paclitaxel (7.03 mM) were obtained from St. Vincent’s University Hospital.

### Proliferation assay

Proliferation was measured using an acid phosphatase-based assay. 1 × 10^3^ cells/well were seeded in 96-well plates, apart from Malme-3M which were seeded at 2 × 10^3^ cells/well. Plates were incubated overnight at 37 °C followed by addition of drug at the appropriate concentrations and incubation for 5 days. All media was removed and the wells were washed once with PBS. 100 μl of freshly prepared phosphatase substrate (7.1 mM paranitrophenol phosphate (Sigma) in 0.1 M sodium acetate (Sigma), 0.1% triton X-100 (BDH), pH 5.5) was added to each well. The plates were incubated in the dark at 37 °C for 1–2 h. The enzymatic reaction was stopped by the addition of 50 µl of 1 M NaOH to each well. The plates were read in a spectrophotometer (BioTek Synergy HT) at 405 nm with a reference wavelength of 620 nm. Growth of drug-treated cells was calculated as a percentage relative to untreated control cells. All assays were performed in triplicate.

### Protein lysate preparation

Protein lysates for melanoma and lung cancer cell lines were prepared from 10 cm dishes seeded with 1 X 10^5^ cells/ml 48 h prior to lysis. Lysis was carried out by addition of 300 μl RIPA buffer (Sigma) with added protease inhibitors (Roche), sodium orthovanadate (Sigma) and PMSF (Sigma). Protein levels in lysates were quantified by the BCA method (Pierce).

### Immunoblotting

Thirty μg of protein lysate from each cell line was separated by SDS gel electrophoresis (Invitrogen). Proteins were transferred to a nitrocellulose membrane using the iBlot semi-dry transfer system (Invitrogen). Blots were incubated overnight in 5% milk/PBS-Tween containing P-gp and MDR3 primary antibody C219 ALX-801-002 (ENZO Life Sciences) (1:1000) or BCRP primary antibody ALX-BXP-21 (ENZO Life Sciences). C219 recognises amino acid sequences (VQEALD and VQAALD) found in P-gp (170 kDA) and MDR3 (140 kDa). ALX-BXP-21 does not cross react with P-gp, MRP-1 or MRP2 according to manufacturer. Primary antibodies were detected using peroxidase-conjugated anti-mouse IgG secondary (1:2000, Sigma). Chemiluminescence was visualized through exposure to Luminol (Santa Cruz) and X-ray film (Sigma).

### Analysis of cancer cell encyclopedia data

The RNA expression of P-gp (ABCB1), BCRP (ABCG2), and MDR3 (ABCB4) were exported from Depmap Portal, data set version: DepMap Public 22Q2 [30]. Drug sensitivity data, expressed as area under the curve (AUC), was exported from the PRISM Repurposing dataset version 19Q4.

### Drug efflux transporter activity assays

PREDEASY™ ATPase Assay Kits (Solvo Biotechnology) were used to measure the effect of docetaxel and ABT-751 on the ATPase activity of drug efflux transporters P-gp and BCRP. Assays were conducted according to the kit instructions. All compounds were dissolved in DMSO, with a starting concentration of 25 µM and 1:2.5 dilutions. Drugs were incubated with membrane suspension for 10 min at 37 °C, before the reaction was blocked, developed and absorbance read at 630 nm. Na_3_VO_4_ insensitive ATPase activity was subtracted to assess transporter specific ATPase activity of the membrane suspension. Data are mean ± SD for each concentration determined in duplicate.

### Statistical analysis

IC_50_ values were calculated using Calcusyn software. Statistical significance was calculated using Student’s *t* test on GraphPad Prism or Microsoft excel. Correlation analysis used Spearman’s rank test calculated using GraphPad Prism. Results were expressed as mean ± SD. *p* values < 0.05 were considered statistically significant.

## Results

### Discrepancies in P-gp, MDR3 and BCRP protein and gene expression in the melanoma cell line panel

**Fig. 1:**
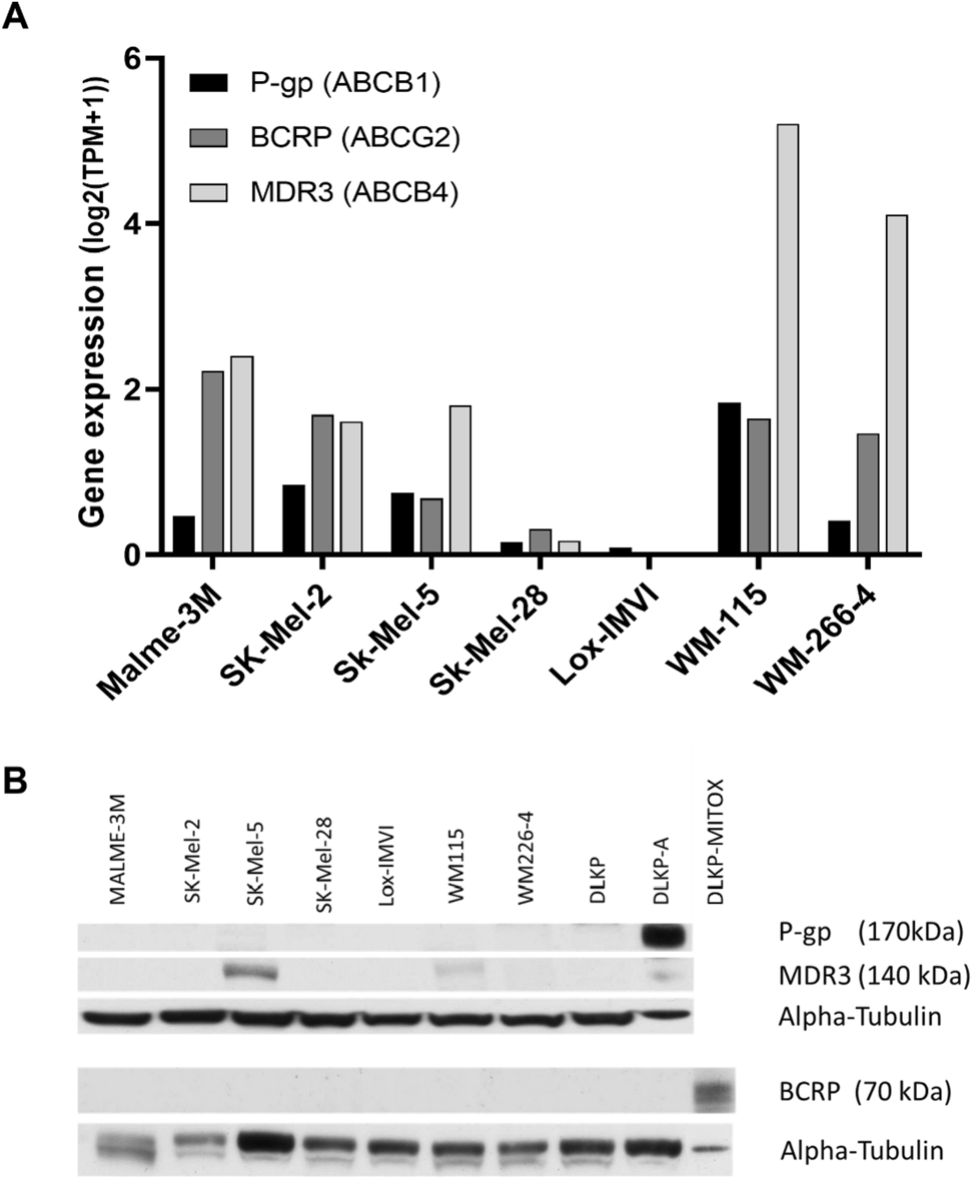
Protein and RNA expression of drug efflux transporters varies across melanoma cell lines in this study. **A** RNA expression of P-gp (ABCB1), BCRP (ABCG2), and MDR3 (ABCB4) for the melanoma cell lines, exported from CCLE Depmap portal. **B** P-gp, MDR3, and BCRP protein expression in the melanoma cell line panel and the control DLKP/DLKP-A/ DLKP-MITOX cell lines. DLKP-A was used as a positive control for P-gp expression. DLKP-MITOX was used as positive control for BCRP detection. Western blot results are representative of three replicates

Expression of P-gp, MDR3, and BCRP was first queried using gene expression data from the Cancer Cell Encyclopedia Depmap Portal [30]. This analysis (Fig. 1A) showed variable RNA expression of the three efflux transporter genes ABCB1 (P-gp), ABCB4 (MDR3) and ABCG2 (BCRP) across the melanoma cell line panel. ABCB4 (MDR3) had the highest expression across the panel, particularly in the WM-115 and WM-244-4 cell lines. WM-115 was also found have the highest expression of ABCB1 (P-gp), while the LOX cell line had extremely low levels of gene transcript for all three transporters.

Follow-up western blot analyses (Fig. 1B), showed the melanoma cell line panel did not express detectable levels of P-gp or BCRP protein suggesting a level of post-transcriptional control for the drug transporter proteins of interest. However, two cell lines, SK-MEL-5 and WM-115 displayed MDR3 protein expression. In contrast, our previous study conducted on melanoma patient tissue samples showed over 80% of samples to express P-gp and/or MRP-1 [13]. This discrepancy suggests a disparity in drug efflux transporter expression in vivo and in vitro. As none of the melanoma cell lines expressed a detectable level of P-gp protein in vitro, DLKP-A (P-gp over-expressing lung cancer cell line) were used as a model of P-gp over-expression [28] and DLKP-MITOX cells were used as a positive control for BCRP protein expression [29].

### ABT-751 is effective at clinically relevant concentrations in melanoma cell lines and is not impacted by drug transporter expression

All melanoma cell lines displayed greater sensitivity to taxanes than ABT-751. Low IC_50_ ranges from 0.07–2.5 nM for docetaxel and 0.32–6.1 nM for paclitaxel were observed, whereas ABT-751 displayed an IC_50_ concentration range of 208.2–1007.2 nM within the melanoma cell line panel (Table 1). WM-115 and WM-266-4 are derived from the primary site and a metastatic site from the same patient [31]. Interestingly, the WM-226-4 cells were significantly more sensitive to docetaxel and paclitaxel compared to WM-115 (*p* < 0.05, Students *t* test) but there was no difference in sensitivity to ABT-751 between the two cell lines (Table 1). There are differences in MDR3 protein expression and P-gp RNA expression levels between these two cell lines, however it is not possible to attribute the variation in drug sensitivity to these factors within this study (Fig. 1B).

**Table 1:** IC_50_ values ± standard deviations for taxanes and ABT-751 in seven melanoma cell lines

Treatment	Sk-Mel-5	Malme-3M	Lox-IMVI	Sk-Mel-28	WM-115	WM-266–4	SK-Mel-2
Docetaxel (nM)	1.3 ± 0.5	2.5 ± 0.3	2.3 ± 0.2	2.2 ± 0.4	1.4 ± 0.2	0.07 ± 0.01^†^	0.32 ± 0.02
Paclitaxel (nM)	3.9 ± 0.6	4.5 ± 0.8	6.1 ± 0.5	5.1 ± 0.2	6.9 ± 1.3	0.32 ± 0.02^†^	1.12 ± 0.06
ABT-751 (nM)	259.7 ± 121.3	465.2 ± 116.1	1007.2 ± 104.5	697.9 ± 115.9	208.2 ± 16.2	298.9 ± 85.5	262.2 ± 92.7

^†^*p* < 0.05 relative to WM-115, using Students *t* test

**Fig. 2:**
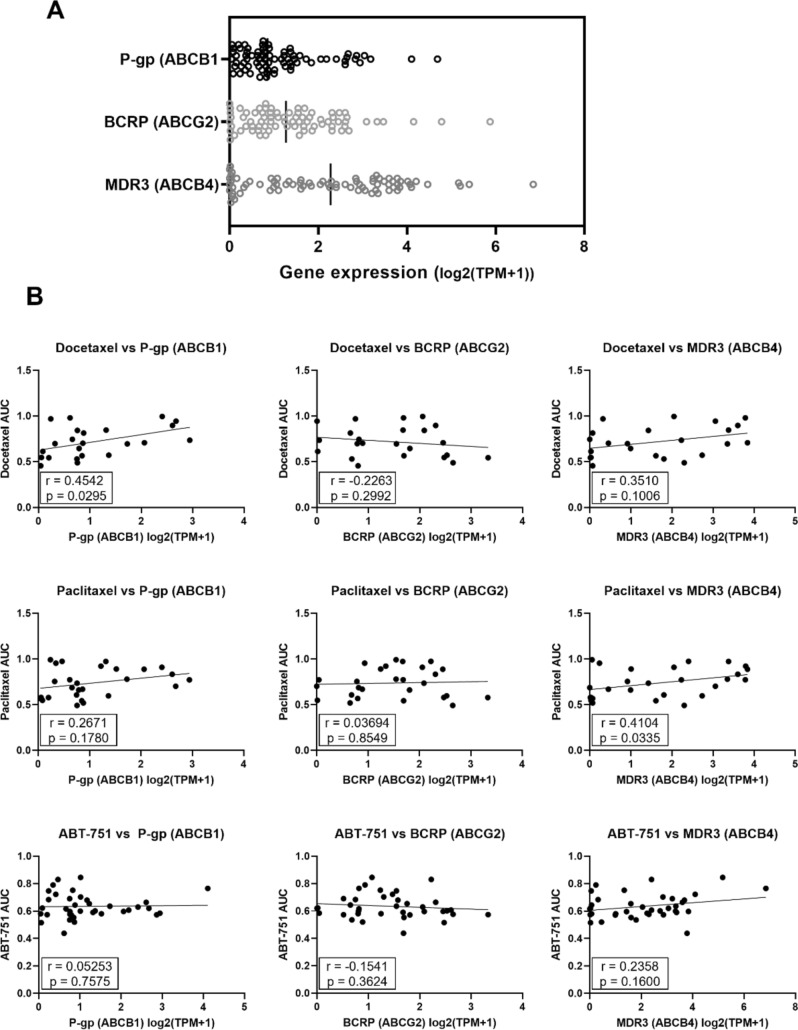
Correlation analysis of drug transporter genes versus drug sensitivity in melanoma cell lines, derived from DepMap Portal CCLE expression and drug sensitivity datasets. **A** Overall gene expression profile of P-gp (ABCB1), BCRP (ABCG2) and MDR3 (ABCB4) in all melanoma cell lines available on CCLE (*n* = 78). **B** Correlation analysis using Spearman’s rank correlation coefficient of MDR drug transporter gene expression versus docetaxel, paclitaxel and ABT-751 drug sensitivity (AUC)

Analysis of RNA expression data from all 78 melanoma cell lines available on the DepMap portal revealed a wide range of gene expression of the three efflux transporters of interest with MDR3 (ABCB4) most highly expressed (Fig. 2A). Comparing this gene expression data to drug sensitivity data from the PRISM Repurposing dataset version 19Q4 available from Depmap portal [30], allowed further examination of efflux transporter gene expression and taxane resistance in a larger sample set. As illustrated in Fig. 2B, moderate (Spearman’s rank correlation coefficient, *r* = 0.4542) and weak (*r* = 0.3510) positive correlations were found between docetaxel toxicity area under the curve (AUC) and P-gp and MDR3 expression, respectively, in melanoma cell lines. This suggests P-gp has influence on taxane efficacy in cell line models, in agreement with a similar screen conducted on 60 cancer cell lines [32]. Some positive correlation was also seen for the sensitivity to paclitaxel and expression of P-gp and MDR3 (*r* = 0.2671 and *r* = 0.4104). No correlation was seen between P-gp expression and response to ABT-751. Overall, BCRP expression had no correlation with sensitivity to the three compounds assessed.

### The anti-proliferative effect of ABT-751 is not impacted by P-gp over-expression

**Fig. 3:**
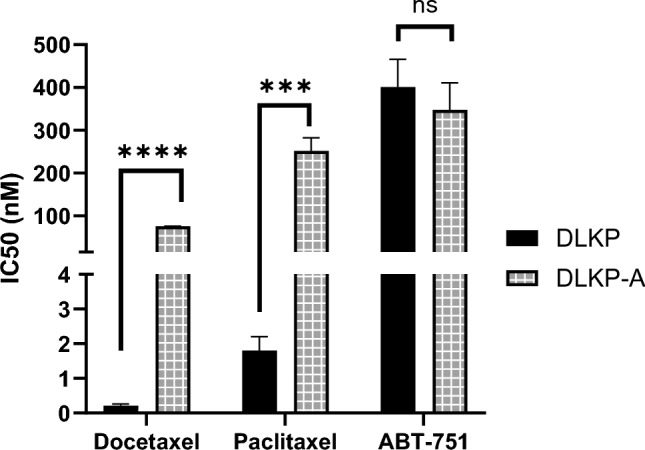
The IC_50_ of docetaxel, paclitaxel and ABT-751 in parental DLKP cells versus P-gp-expressing, MDR DLKP-A. Difference between cell lines was assessed using unpaired *t* test, ****p < 0.0001, ***p < 0.001

Following the suggestion that ABT-751 efficacy is not influenced by drug transporter expression (Fig. 2B), we next tested the correlation experimentally, using a previously characterized cell line model of P-gp-mediated MDR. The P-gp over-expressing DLKP-A cell line displayed 139-fold resistance (*p* < 0.0001) to paclitaxel and 358-fold resistance to docetaxel (*p* < 0.001) compared to the P-gp-null parent cell line DLKP (Fig. 3). In contrast, there was no significant difference in ABT-751 IC_50_ between DLKP and DLKP-A (*p* = 0.4007), supporting the data in Fig. 2B.

### Elacridar impacts ABT-751 response in MDR3-expressing SK-MEL-5 cell line

To confirm the observed resistance of the DLKP-A cell line to taxanes is driven by P-gp drug transporter activity, co-treatments with the P-gp/BCRP inhibitor elacridar were carried out in the parental DLKP and resistant DLKP-A cell lines. Low concentrations of docetaxel were used to treat the DLKP (0.2 nM) and DLKP-A (10 nM) cell lines to produce < 10% cytotoxicity and provide a window to observe increased cytotoxicity due to efflux pump inhibition. Elacridar significantly enhances docetaxel cytotoxicity in DLKP-A, and reverses the acquired resistance phenotype (Table 2).

**Table 2:** The effect on relative cell proliferation following addition of 500 nM elacridar to docetaxel treatment in parental DLKP and resistant DLKP-A cell lines

Cell line	Docetaxel concentration (nM)	% cell proliferation ± StdDev			*p* value
		Docetaxel	500 nM elacridar	Combined agents	
DLKP	0.2	89.9 ± 2.9	102.6 ± 0.6	85.5 ± 1.8	0.154
DLKP-A	10	98.7 ± 0.3	97.5 ± 0.8	0.77 ± 0.3	< 0.001*

Values represent % proliferation ± standard deviation of triplicate experiments. A Student’s *t* test was used to compare docetaxel only with the combined agents values

*Significant, *p* < 0.05

Following the impact of elacridar on docetaxel toxicity in DLKP-A, ABT-751 and P-gp substrate paclitaxel [33] were combined in DLKP-A, resulting in a significant increase in toxicity (Table 3). While additive mechanistic effects of paclitaxel and ABT-571 cannot be discounted, this suggests that P-gp-mediated paclitaxel transport may be impeded by ABT-751. ABT-751/elacridar combination assays were also carried out in two melanoma cell lines, LOX-IMVI (P-gp/BCRP/MDR3-null by RNA and protein expression), and SKMEL-5, which displayed the highest MDR3 protein expression of the cell lines investigated (Fig. 1). As with the docetaxel combinations in Table 2, a low concentration of ABT-751 (150–250 nM) was used that resulted in 5–10% cytotoxicity, leaving a window to observe additional toxicity associated with potential efflux pump inhibition. Elacridar has been reported to inhibit MDR3 [34]. As detailed in Table 3, no effect was seen upon addition of elacridar to ABT-751 in LOX-IMVI but in the SKMEL-5 cell line, the addition of elacridar caused a small but significant (*p* = 0.020, Student’s *t* test) decrease in cell proliferation compared to ABT-751 alone.

**Table 3:** Combination proliferation assays combining ABT-751 with elacridar (500 nM) or paclitaxel (250 nM) in the drug-resistant P-gp + /MDR3 + DLKP-A, the P-gp − /MDR3 − LOX-IMVI cell line, and the P-gp − /MDR3 + SK-MEL-5 cell line

Cell line	ABT-751 concentration (nM)	% cell proliferation ± StdDev				*p* value
		ABT-751	500 nM elacridar	250 nM paclitaxel	Combined agents	
DLKP-A	150	95.6 ± 0.5	–	86.9 ± 2.5	78.9 ± 3.6	0.030*
LOX-IMVI	250	92.3 ± 7.0	101.2 ± 1.7	–	92.5 ± 8.8	0.984
SK-MEL-5	250	90.1 ± 0.9	100.2 ± 0.2	–	81.9 ± 3.6	0.020*

Values represent % proliferation ± standard deviation of triplicate experiments. A Student’s *t* test was used to compare ABT-751 with the combined agents’ values

*Significant, *p* < 0.05

**Fig. 4:**
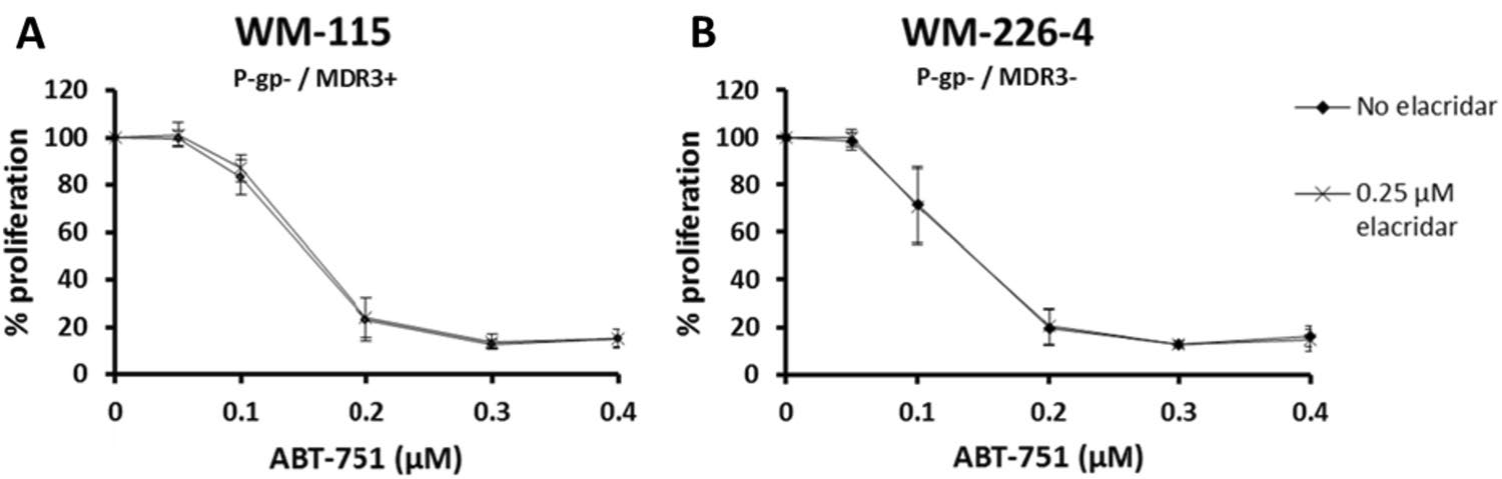
IC_50_ ABT-751 proliferation assays carried out in the presence or absence of 0.25 μM elacridar in **A** the P-gp −/MDR3 + WM-115 and **B** P-gp −/MDR3−  WM-266–4. Error bars represent the standard deviation of triplicate experiments, *significant, *p* < 0.05

Two further melanoma cell lines were treated with ABT-751 in the presence and absence of elacridar. Elacridar had no significant effect when combined with ABT-751 in the P-gp-negative (−), MDR3 −  WM-266-4 cell lines, as expected, or in the P-gp −, MDR3-positive (+) WM-115 cell line (Fig. 4). The WM-115 cell line expresses lower levels of MDR3 than SK-MEL-5 (Fig. 1).

### ABT-751 is a potential BCRP substrate and P-gp inhibitor

**Fig. 5:**
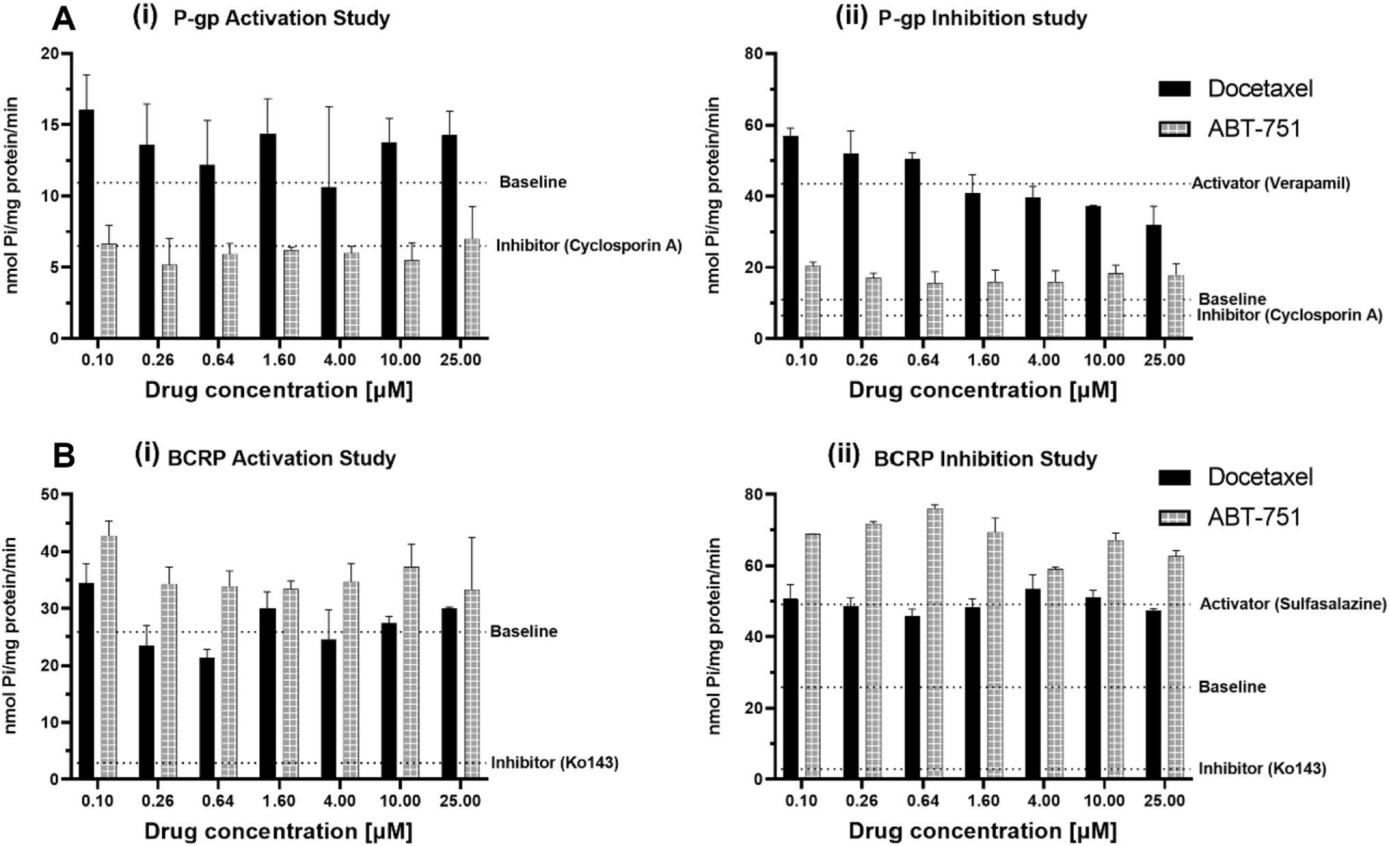
P-gp **A** and BCRP **B** ATPase activity assays in the presence of docetaxel and ABT-571. The baseline activity represents the ATPase activity measured in the absence of added test compounds. For activation studies, **A** (i) and **B **(i), control inhibited transporter activity was induced by cyclosporine A (P-gp) or Ko143 (BCRP). For inhibition studies, **A** (ii) and **B** (ii), maximal ATPase activity was induced by verapamil (P-gp) or sulfasalazine (BCRP). Data are mean + / − SD for each concentration determined in duplicate

Following the proliferation assays in Fig. 4 and Table 3, we next conducted ATPase assays to directly gauge the activation or inhibition of P-gp and BCRP ATPase activity by ABT-751. No MDR3 ATPase assays were available. Cell-free membrane preparations of P-gp and BCRP were exposed to varying concentrations of docetaxel (a known P-gp substrate) and ABT-751 with the measured changes in liberated inorganic phosphate (Pi) as the transporters consume ATP conferring a read-out of transporter activity. P-gp substrate docetaxel increased the activity of P-gp ATPase above baseline for 6/7 concentrations tested but in contrast, ABT-751 decreased P-gp ATPase activity below baseline for all concentrations, similar to known P-gp inhibitor Cyclosporin A (Fig. 5A(i)). The ability of docetaxel and ABT-751 to inhibit substrate-activated P-gp ATPase activity was also investigated (Fig. 5A(ii)). As a P-gp substrate, docetaxel further increased P-gp ATPase activity in the presence of verapamil at lower concentrations, decreasing P-gp ATPase activity above 1.6 µM in a concentration dependent manner. However, ABT-751 consistently inhibited verapamil-activated P-gp ATPase activity at all concentrations but did not return P-gp ATPase activity to baseline, or match the ability of cyclosporin A to inhibit verapamil-activated P-gp ATPase activity (Fig. 5A(ii)). This suggests that some active P-gp-mediated substrate transport may remain ongoing in the presence of ABT-751. ABT-751 does interact with P-gp, but acts in an inhibitory fashion, decreasing the rate of ATP consumption by the active transporter, both from baseline and for pre-activated P-gp.

The ATPase profile for ABT-751 is considerably different in the BCRP ATPase assays. Docetaxel is not a BCRP substrate and it had little effect on BCRP ATPase from baseline (Fig. 5B (i)). ABT-751 does appear to interact with BCRP, increasing BCRP ATPase activity above baseline at all concentrations examined (Fig. 5B(i)). For BCRP pre-activated with sulfasalazine, docetaxel had little to no effect on rates of liberated Pi while ABT-751 further enhanced BCRP ATPase activation (Fig. 5B(ii)). Both BCRP ATPase studies suggest ABT-751 can activate BCRP ATPase and is therefore a potential BCRP substrate.

## Discussion

For metastatic melanoma patients whose tumours do not express targetable markers, treatment options are limited to chemotherapies with poor response rates and associated toxicities [3]. Taxane-based therapies show potent cytotoxic effects in vitro, however when tested in patients responses are limited, even in combination chemotherapeutic regimes [7]. One potential explanation for the lack of efficacy of taxanes in melanoma is expression of drug efflux transporters like P-gp [35]. ABT-751 is a novel oral anti-mitotic agent which is not reported to be a substrate of P-gp, and therefore may overcome P-gp-mediated drug resistance in melanoma [26]. Our study confirms that ABT-751 is active against melanoma cell lines at physiologically relevant levels, it inhibits P-gp ATPase activity, and it may be a BCRP and/or MDR3 substrate.

Our initial exploration of drug efflux transporter protein expression in melanoma cell lines elicited differential results. Gene expression analysis based upon CCLE data, showed a number of the melanoma cell lines express ABCB1, ABCB4 and ABCG2 transcripts, however levels varied widely. Western blot analysis revealed few of the melanoma lines tested had detectable levels of protein, with the exception of SK-MEL-5 and WM-115, which both expressed MDR3. Variable levels of P-gp expression in melanoma tissue and cell lines are reported in the literature [12, 36, 37]. A previous study from our laboratory detected P-gp protein in over 80% of melanoma tissue samples [13]. Such discrepancy in P-gp levels between tumour tissue and cell lines is not unusual, and could be attributed to loss of expression upon generation of immortalised cell lines, or transporter expression being induced in response to tumour micro-environment or drug treatment [38, 39]. Expression of P-gp in melanoma has been linked to disease progression and an aggressive phenotype in patient tissue samples [13] and in vitro models [40]. More physiologically relevant models that better recapitulate the expression profile of melanoma tissue in vitro are needed.

Comparing the toxicity of traditional taxanes to ABT-751, we found melanoma cell lines were more sensitive to taxanes, with lower IC_50_ values observed for both docetaxel and paclitaxel compared to ABT-751 in all seven cell lines tested. Nevertheless, the IC_50_ values for ABT-751 in the melanoma cell lines are within the achievable serum concentration range of the drug, 1.35–4.04 μM (Table 1) [20]. As levels of P-gp expression were low or absent in the seven melanoma cell lines used, we utilized the P-gp-over-expressing cell line model DLKP-A to assess whether ABT-751 is a substrate of P-gp. DLKP-A did not display significant resistance to ABT-751 compared to parental P-gp-null cell line DLKP, while docetaxel and paclitaxel experienced > 130-fold resistance in DLKP-A compared to DLKP (Fig. 3). This is in agreement with previous studies that found over-expression of multi-drug resistance-associated transporters, including P-gp, did not confer resistance to ABT-751 in cell line and xenograft models [26, 27, 41] and aligns with the ATPase assay results in Fig. 5.

When elacridar was added to ABT-751, a significant but limited anti-proliferative effect occurred in the MDR3-expressing SK-MEL-5 cell line (Table 3). This effect did not occur in the only other MDR3-expressing melanoma cell line examined (WM-115), potentially explained by lower expression levels of MDR3 at the protein level (Fig. 1). Although elacridar is generally classed as a P-gp inhibitor, a recent study using P-gp knockout/MDR3-expressing cell lines has shown elacridar and several other known P-gp inhibitors such as valspodar and gefitinb can function as MDR3 inhibitors [34]. For this reason, we hypothesise the observed increase in ABT-751 response when elacridar is added in SK-MEL-5 cells, may be attributed to interaction between ABT751 and elacridar when a sufficiently high level of MDR3 is present to impact proliferation at the drug concentrations tested. The correlative analysis of publicly available data in Fig. 2 does hint at a potential relationship between MDR3 expression and ABT-751 sensitivity at the transcriptomic level but as the results in Fig. 1 show, RNA levels of drug transporters may not translate to protein levels. As this study only examined three potential drug efflux transporters, it is also important to note that there are additional active and passive drug transporters that could be mediating the observed effects [42]. Further in-depth study of MDR3 protein in relation to ABT-751 transport is warranted.

P-gp and BCRP ATPase assays revealed that ABT-751 can inhibit P-gp ATPase activity and activates BCRP ATPase activity, potentially acting as a BCRP substrate (Fig. 5). ATPase assays only report on the ability of compounds to modulate transporter activity, transport (accumulation or efflux) assays would be required to provide definitive confirmation of ABT-751 transport by BCRP. The inhibitory action of ABT-751 on P-gp may explain the additional anti-proliferative effect observed when combined with paclitaxel in DLKP-A cells (Table 3). Furthermore, our finding is in agreement with a study by Frost et al., who reported pairwise synergy between ABT-751 and docetaxel in a variety of pre-clinical models [43].

This study provides evidence that ABT-751 has potential in combination chemotherapeutic regimens for P-gp + MDR cancers. A significant trend in anti-cancer drug development is the search for therapeutic compounds that are not substrates of drug efflux transporters. This work has shown ABT-751 is not a substrate of P-gp and may inhibit the action of P-gp. Although only displaying modest activity at clinical trial, it is evident that taxane alternative drugs such as ABT-751, upon further refinement and development, may offer therapeutic options in cases of chemotherapy resistant cancers. In fact, recent advances have been made in the development of ABT-751 derivatives with increased in vitro and in vivo cytotoxicity compared to the parental compound [44]. Furthermore, given the inhibitory characteristics of ABT-751, particular value could be found in combination with P-gp substrate BRAF and MEK inhibitors approved for melanoma [9, 11]. These findings are also important in the context of brain metastasis, which develops in almost 50% of all advanced melanoma cases [45]. Many chemotherapies are prevented from crossing the blood–brain barrier by high expression of efflux transporters, thus ABT-751 warrants further investigation as a compound that can cross, or facilitate the crossing of partner compounds, into brain tissue. Further testing of ABT-751 in in vitro and in vivo models of P-gp + melanoma are required to provide further rationale for ABT-751 as a viable candidate for the treatment of MDR melanoma.

## Data Availability

Data available upon reasonable request to corresponding author.
